# The Recent Heavy Rush of Work at Cambridge

**Published:** 1914-11-21

**Authors:** 


### ACCOMMODATION PROBLEM FOR THE WOUNDED.

### The Recent Heavy Rush of Work at Cambridge.

No fewer than five hundred wounded soldiers arrived in Cambridge the week before last. Thus the physicians, surgeons, anæsthetists, matrons, sisters, and nurses at the 1st Eastern General Hospital have been called upon to undertake some very hard work, which they have done with great cheerfulness and devotion to duty. The Commanding Officer and his staff have also been heavily taxed in preparing accommodation.

A very large number of wounded soldiers, both British and Belgian, were landed at Southampton on November 1. From this batch of wounded it was arranged to send about 111 to the 1st Eastern General Hospital, Cambridge. These were dispatched in a Midland Railway hospital train, which brought these 111 Belgians direct from the great battlefield around Ostend. Among them were three officers; these were conveyed in an ambulance to the Research Hospital, Hills Road, Cambridge, and the remainder were transferred in motor ambulances and cars to the 1st Eastern General Hospital. Refreshments were prepared in readiness for the wounded upon their arrival at Cambridge by the Women's Voluntary Aid Detachment No 4, under the direction of their Commandants, Mrs. Widdicombe and Miss Bishop. The ambulances and cars used for conveying the wounded to the hospital were under the supervision of Mr. A. G. Gamble, of Cambs. No. 7 Voluntary Aid Detachment.

Those who were on the platform and who assisted in the arrangements, which were so admirably carried out, included Colonel J. Griffiths, R.A.M.C., M.D., M.S., F.R.C.S.; the Rev. C. F. Townley, M.A., County Director British Red Cross Society; Mr. J. C. Lawson, Borough Director; Lieutenant Porter, Sergeant Coe, and the stationmaster (Mr. Ablitt) and his superintendent, Mr. FitzJohn.

One of the three wounded Belgian officers who were conveyed to the Research Hospital was recognised as one of those who a year ago or thereabouts played for the Belgians against the Cambridge University hockey team. He appears to have been in the siege of Antwerp, and had been constantly in action from that time onward until in the district of Ypres he received wounds from a shell in both legs, whilst a number of his comrades were fatally wounded. This wounded Belgian officer was full of praise for the British and Indian soldiers.

The following Tuesday another hospital train arrived with about 150, and among them were many of the 6th Division encamped but a short time ago in Cambridge. There were a large proportion of "stretcher" cases. The various waiting-rooms had been suitably arranged for their reception, and refreshments prepared in a remarkably quick and capable manner, to the great comfort of the recipients. The usual arrangements at the station were carried out in a most efficient way by the members of the Cambs. No. 44 Voluntary Aid Detachment and Cambs. No. 32 Voluntary Aid Detachment, under their respective Commandants, Miss Warrington, of Waterbeach Vicarage, and Mrs. Goodchild. The Cambs. No. 13 Men's Voluntary Aid Detachment, under the command of Mr. W. B. Jenyns, also rendered very valuable help.

Postal arrangements were made for the convenience of the wounded, and these were carried out by Miss B. Wherry, Miss Townley, and Mrs. Bidwell.

But a very short time had elapsed when on Friday evening, November 6, another hospital train arrived, bringing about 130 wounded Belgian soldiers. The various arrangements were most efficiently carried out by the Cambs. No. 36 Voluntary Aid Detachment from Cottenham, consisting of the following members under the direction of Commandant Mrs. Hayden Cox, Lady Superintendent-Nurse Chambers, Quartermaster Mrs. Ada Bryant, Assistant-Quartermaster Miss E. Leader, Assistant-Commandant Miss E. Leader, and Dr. Hayden Cox, medical officer. The stretcher-bearer detachment from Histon also rendered invaluable help. Cambs. No. 7 Voluntary Aid Detachment was under the command of Dr. A. Wood, assisted by Quartermaster Scott.

### X-RAY WORK AT ADDENBROOKE'S.

With great promptitude most of these hundreds of wounded British and Belgian soldiers were sent to Addenbrooke's Hospital to be subjected to *x*-rays. In connection with this valuable work Dr. Shillington Scales, who is in charge of the electrical and *x*-ray department, has been ably assisted by the pharmacist of the hospital, Mr. W. J. Field, who has, with great devotion, ungrudgingly laboured into the late hours of the night for the benefit of these wounded soldiers.

### ANOTHER RED CROSS HOSPITAL.

The houses in Hills Road, Cambridge, until recently known as Cheshunt College, have been converted into a Red Cross hospital or home for the reception of Belgian wounded, which acts as a clearing-house for the 1st Eastern General Hospital. The building has been loaned free of rent by Mr. Holloway, and the Cambs. 8th and 28th Voluntary Aid Detachments have equipped and furnished the home, accomplishing their task at only a few hours' notice. The accommodation consists of twenty-one well-ventilated and airy bedrooms, a very large sitting-room, well furnished, where various games are provided, and dining-rooms where those who are able take their meals. The home is under the direction of Mr. W. Mirless. Captain James, late of the Church Army Home, is assisting Mr. Mirless, and Mrs. James is acting as housekeeper. At present there are about forty patients at the home.

